# Type II and III Selective Fetal Growth Restriction: Perinatal Outcomes of Expectant Management and Laser Ablation of Placental Vessels

**DOI:** 10.6061/clinics/2018/e210

**Published:** 2018-04-17

**Authors:** Mariana Yumi Miyadahira, Maria de Lourdes Brizot, Mário Henrique Burlacchini de Carvalho, Sckarlet Ernandes Biancolin, Rita de Cássia Alam Machado, Vera Lúcia Jornada Krebs, Rossana Pulcineli Vieira Francisco, Cleisson Fábio Andrioli Peralta

**Affiliations:** I Departamento de Ginecologia e Obstetricia, Faculdade de Medicina (FMUSP), Universidade de Sao Paulo, Sao Paulo, SP, BR; II Departamento de Pediatria, Faculdade de Medicina (FMUSP), Universidade de Sao Paulo, Sao Paulo, SP, BR; III Gestar Medicina e Cirurgia Fetal, Sao Paulo, SP, BR; IV Hospital do Coracao, Sao Paulo, SP, BR

**Keywords:** Twin Pregnancy, Monochorionic Pregnancy, Selective Fetal Growth Restriction, Expectant Management, Laser Ablation of Placental Vessels, Perinatal Outcomes

## Abstract

**OBJECTIVES:**

To describe the perinatal outcomes of type II and III selective fetal growth restriction (sFGR) in monochorionic-diamniotic (MCDA) twin pregnancies treated with expectant management or laser ablation of placental vessels (LAPV).

**METHODS:**

Retrospective analysis of cases of sFGR that received expectant management (type II, n=6; type III, n=22) or LAPV (type II, n=30; type III, n=9). The main outcomes were gestational age at delivery and survival rate.

**RESULTS:**

The smaller fetus presented an absent/reversed “a” wave in the ductus venosus (arAWDV) in all LAPV cases, while none of the expectant management cases presented arAWDV. The median gestational age at delivery was within the 32nd week for expectant management (type II and III) and for type II LAPV, and the 30^th^ week for type III LAPV. The rate of at least one twin alive at hospital discharge was 83.3% and 90.9% for expectant management type II and III, respectively, and 90% and 77.8% for LAPV type II and III, respectively.

**CONCLUSION:**

LAPV in type II and III sFGR twins with arAWDV in the smaller fetus seems to yield outcomes similar to those of less severe cases that received expectant management.

## INTRODUCTION

Selective fetal growth restriction (sFGR) complicates 12-25% of monochorionic-diamniotic (MCDA) twin pregnancies 1. sFGR is defined as an estimated fetal weight (EFW) below the 10^th^ percentile for at least one twin and an EFW discrepancy between the twins ≥25% 2. Although the etiology of this condition is not completely understood, unequal placental sharing combined with different types of placental anastomosis between the twins appears to play a key role in the clinical manifestations and outcomes 1,3-5.

A classification system based on umbilical artery (UA) Doppler flow of the smaller twin was proposed by Gratacós et al. 1: in type I, there is positive end-diastolic flow in the UA (pEDFUA); in type II, there is persistent absent/reversed (ar) EDFUA; and in type III, there is intermittent absent/reversed (iar) EDFUA 3. Type I cases have a good prognosis and can be managed expectantly 6,7. Type II and III cases carry a high risk of fetal/perinatal mortality and postnatal handicap for both twins when managed expectantly. In these cases, laser ablation of placental vessels (LAPV) and cord occlusion of the smaller twin have been proposed as management options 1,8-11. Cord occlusion is an option when selective reduction of the smaller twin is approved by the parents and allowed by local regulations. Otherwise, LAPV is available when cord occlusion is not possible for any reason, but there is a paucity of information about whether compared to expectant management, it improves perinatal outcomes.

In Brazil, selective termination of twins by cord occlusion in cases of sFGR is not legal. Therefore, LAPV is the only alternative to expectant management and has been offered in some centers in the last six years. The aim of the present study was to present the perinatal outcomes of type II and III sFGR cases that received expectant management or LAPV.

## MATERIALS AND METHODS

This was a retrospective study of all cases of sFGR managed expectantly or treated by LAPV at the São Paulo University (USP) Medical School and at the Heart Hospital, São Paulo, Brazil, from 2007 to 2016. The study was approved by the Institutional Review Board of USP (1.754.515).

The inclusion criteria were as follows: MCDA twin pregnancy with type II or III sFGR diagnosed before 26 weeks of gestation, diagnosis and antenatal management at one of the participating centers, no fetal or neonatal malformations, and a cervical length of at least 15 mm (the 5^th^ percentile according to To et al. 12) before the LAPV procedure. Patients with twin oligo-polyhydramnios sequences (TOPS) or twin anemia-polycythemia sequences (TAPS) were not included.

Chorionicity and amnionicity were determined on the first trimester scan or on placental histological examination after birth. sFGR was defined as an EFW below the 10^th^ percentile of local growth charts 13 for at least one twin and an EFW discordance ≥25% 2. EFW discordance was calculated as the difference between the EFW of the larger and the smaller fetus divided by the EFW of the larger fetus, and the results are expressed as a percentage. The type of sFGR was defined according to the classification by Gratacós et al. 1.

Ultrasound and Doppler examinations were performed transabdominally with a 3.5-5.0 MHz curvilinear-array transducer (Envisor-Phillips; Voluson Expert E8, General Electric Healthcare; UGEO WS80a, Samsung; Accuvix XG, Samsung) by operators with considerable experience in examining twins. Doppler waveforms of the UA were assessed with a minimum of three measurements on a free loop, avoiding maternal and fetal breathing and movement.

All patients in the expectant management group received antenatal care at the Multiple Pregnancy Unit of the Obstetrics Department at USP Medical School, where MCDA pregnancies are examined fortnightly up to 26 weeks of gestation to detect TOPS, TAPS and sFGR. Pregnancies with a type II or III UA pattern were followed twice a week until 26 weeks of gestation. Since 2012, LAPV has been offered to patients with an absent/reversed “a” wave in the ductus venosus (arAWDV). At a gestational age (GA) ≥26 weeks, hospital admission was considered for daily fetal monitoring in cases of type II sFGR. For type III cases, ultrasound monitoring was performed twice a week in the outpatient clinic. Delivery was indicated in the presence of arAWDV, abnormal biophysical profile, fetal heart rate traces or persistent reverse end diastolic flow by UA Doppler. Corticosteroid therapy was administered when fetal deterioration was apparent.

The intervention (LAPV) group comprised patients who underwent ultrasound evaluations and were treated by a single operator (CFAP) at the Fetal Medicine and Surgery Center (Gestar) and at the Heart Hospital. Since 2008, endoscopic LAPV has been offered for the treatment of type II and III sFGR with arAWDV before 26 complete weeks of gestation. All patients who underwent the laser procedure signed an informed consent. After the administration of loco regional anesthesia, the LAPV procedure was performed using a diode laser source (Medilas D’Skinpulse, Dornier MedTech, Germany) at 30 to 40 W with a 1.2-mm semi-rigid fiber optic endoscope (11530 AA, Karl Storz, Germany), straight sheaths for posterior placentas (11530 KA or 11530 KC, Karl Storz), curved sheaths for anterior placentas (11530 KB or 11530 KC, Karl Storz), a trocar (11650 TG, Karl Storz) and a 10 French cannula (Performa, Cook, Belgium) for use with the 11530 KC sheaths. The procedure was conducted as follows: the placental chorionic plate vessels through the amniotic cavity of the larger fetus were initially mapped endoscopically; the vascular equator (the expected location of most arterio-venous anastomoses) was identified; and an ablation line on the chorionic plate was created from one edge of the placenta to the other, including arterio-venous anastomoses and vessels with unknown courses (such as those crossing the inter-twin membrane from the larger twin to the growth-restricted twin). Caution was taken to preserve vessels originating from and returning to the same fetus that were surrounded by this ablation line. The cauterization process was performed sufficiently slowly to create a visible white line on the surface of the placenta. There was no predefined minimum distance from the coagulation line to the vessels that needed to be preserved. After the intervention, patients remained in the hospital at least for 12 hours to rest and received oral Nifedipine (20 mg, 8/8 hours) and analgesics according to individual need.

Patients who underwent LAPV had their followups and deliveries at the referring centers. Most cases in the expectant management group delivered at USP Medical School.

Demographic characteristics of the mothers, ultrasound and perinatal data, and information about the neonates up to hospital discharge were obtained from medical records.

Perinatal outcomes of the expectant management and LAPV are presented for type II and type III sFGR groups. The main outcomes considered are gestational age at delivery and survival rate. Perinatal outcomes are also presented according to the larger and smaller twins.

Means and standard deviation (SD) or medians and range were used to describe continuous variables, and absolute and relative frequencies were calculated to describe the categorical data.

Comparisons between expectant management and LAPV treatment were not performed due to the unequal frequency of arAWDV between the groups, which is the main indicator of LAPV.

## RESULTS

A total of 67 MCDA twin pregnancies with sFGR type II (n=36) or type III (n=31) were included. In total, 83% (30/36) of the type II cases and 29% (9/31) of the type III cases underwent LAPV. Table 1 presents the baseline characteristics of the study population according to the type of sFGR and the management strategy. The smaller twin presented with arAWDV in all the cases that underwent LAPV, whereas none of the fetuses in the expectant management group presented this finding at diagnosis.

Perinatal outcomes according to management strategy are presented in Table 2. The overall fetal death rate was 25.4% (34/134), with a rate of 30.6% (22/72) for type II sFGR cases and 19.4% (12/62) for type III sFGR cases.

Premature rupture of membranes <32 weeks and <34 weeks occurred in 17.14% (6/35) and 28.57% (10/35), respectively, of the cases treated by LAPV. Overall, the main reasons for delivery in the LAPV and expectant management groups were preterm premature rupture of membranes, 40% (14/35) *versus* 0% (0/27); abnormal fetal wellbeing, 34.3% (12/35) *versus* 70.4% (19/27); threatened preterm labor, 8.6% (3/35) *versus* 7.4% (2/27); death of one fetus, 5.7% (2/35) *versus* 14.8% (4/27); placental abruption, 2.9% (1/35) *versus* 0% (0/27); and spontaneous labor ≥34 weeks, 8.6% (3/35) *versus* 7.4% (2/27).

## DISCUSSION

In the present study, we described the perinatal outcomes of LAPV and expectant management for MCDA twin pregnancies with type II and III sFGR. The findings demonstrate that both management strategies seem to yield similar perinatal outcomes, provided that LAPV is adequately indicated and performed by an experienced operator and that expectant management cases are closely monitored. Our results support previous findings on expectant and laser management strategies for type III sFGR 9. Recently, Peeva et al. 10 have presented the outcomes of 142 type II sFGR pregnancies subjected to laser photocoagulation of placental communicating vessels, and their data corroborate our findings regarding laser treatment in cases of type II sFGR.

Three management options are available for type II and III sFGR: expectant management, regardless of the severity of the hemodynamic compromise in the smaller twin; LAPV, or cord occlusion of the smaller twin, regardless of the DV flow pattern at diagnosis; and LAPV, or cord occlusion of the smaller twin, only in cases of arAWDV. As mentioned above, cord occlusion for selective feticide in cases of sFGR is not an option in several countries. Moreover, there are not sufficient data to support any of the abovementioned management options, and no randomized trials are currently ongoing to elucidate this issue. Therefore, our study may be useful, as it provides additional information on perinatal outcomes in cases of sFGR managed according to the first or third options described above.

We acknowledge that the groups in the present study are not comparable, as all the cases in the LAPV group had arAWDV at diagnosis, while none of the cases in the expectant management group had this finding. However, by demonstrating similarity in outcomes between the two groups, one could conclude that LAPV in the presence of severely abnormal DV flow prior to 26 weeks gestation at least yielded results similar to those obtained with expectant management of less severe cases. As arAWDV is an ominous finding, it is very unlikely that a randomized trial comparing LAPV to expectant management in these cases diagnosed before viability will be conducted. Considering the high risk of death of the smaller twin, it is highly unlikely that parents will choose expectant management.

One potential limitation is the number of cases of each management strategy based on sFGR type, with fewer expectant management cases in the type II sFGR group and fewer LAPV cases in the type III sFGR group. It is possible that selection bias occurred, considering that all expectant management cases came from the National Health System (NHS), and first trimester scans are not routinely performed for these patients. The few type II sFGR cases that were referred to USP Medical School Hospital consisted of patients who had the chance to undergo an early scan and probably presented with a less severe fetal compromise. The patients who underwent LAPV predominantly had insurance or were receiving private care and thus were followed beginning in the first trimester with an adequate ultrasound schedule for twins. Additionally, as demonstrated by others, type II sFGR is the most severe, with a shorter latency from diagnosis to fetal death or delivery compared to type III sFGR 1. It is possible that several patients under NHS care suffered a miscarriage before referral to USP Medical School Hospital. The number of type III sFGR cases that underwent LAPV was limited because this procedure was only indicated upon identification of arAWDV, and the latency to fetal deterioration is longer compared to that for cases of type II sFGR 1,3. This limitation also explains why only two patients underwent LAPV at the USP Medical School Hospital during the study period. These two cases were not included in the present analysis because LAPV was performed by a different operator. It was not the purpose of the present study to compare the perinatal outcomes of type II and type III UA Doppler patterns. Nevertheless, the perinatal outcomes for both Doppler patterns were similar to those of previous studies, with a worse prognosis for type II cases 1,6,8,14,17. Specific comparisons between studies 1,6,8,14,17 are difficult due to heterogeneity in data presentation, laser indication and type of intervention performed (laser or cord occlusion). Moreover, due to the scarcity of these cases and the difficulties in performing randomized studies, a meta-analysis of individual patient data is a reasonable option to obtain sound evidence.

The main finding of this study is that in cases of type II and III sFGR with arAWDV of the smaller twin, LAPV seems to yield outcomes similar to those of expectant management for less severe sFGR cases (without arAWDV of the smaller twin). This finding may help in cases with arAWDV, in those situations where patients are reticent about invasive procedures, to decide toward the LAPV.

## AUTHOR CONTRIBUTIONS

Miyadahira MY developed the project, collected data, participated in data analysis and wrote the manuscript. Brizot ML developed the project, participated in data analysis and wrote the manuscript. Carvalho MH participated in the manuscript revision and contributed to the discussion. Biancolin SE participated in data collection and analysis. Machado RC participated in data collection and analysis. Krebs VL participated in data collection and analysis and manuscript review. Francisco RP revised the manuscript and contributed to the discussion. Peralta CF performed the procedure of laser ablation of placental vessels, collected data, and wrote and revised the manuscript.

## Figures and Tables

**Table 1:** Baseline characteristics of the study population of monochorionic twin pregnancies with type II or III Doppler patterns according to management strategy (expectant management or laser ablation of placental vessels).

Characteristics	Expectant		Laser	
	Type II, n=6	Type III, n=22	Type II, n=30	Type III, n=9
**Maternal**				
Age, years	24.95 (17-34.2)	30.6 (17-45.7)	30 (15-39)	29 (18-38)
White ethnicity	3/6 (50)	17/22 (77.3)	19/30 (66.3)	8/9 (88.9)
Nulliparous	4/6 (66.7)	13/22 (59.1)	15/30 (50)	2/9 (22.2)
**Pregnancy/ultrasound**				
GA at diagnosis/therapy, weeks	20 (16.43-22.57)	20.42 (16.57-26)	21.64 (17.86-25)	22.29 (17.57-26)
Severe cases*	5/6 (83.3)	18/22 (81.8)	30/30 (100)	9/9 (100)
Abnormal ductus venosus	0/6	0/22 (0)	30/30 (100)	9/9 (100)
EFW discordance at diagnosis/Laser	38.67 (4.52-57.27)	29.36 (1.69-46.7)	35.39 (25.35-61.73)	36.42 (27.27-40.65)

Data are presented as median (minimum-maximum) or number (%). GA, gestational age; EFW, estimated fetal weight. *Severe cases were defined by the presence of any of the following criteria: early onset (i.e., GA <22 weeks), inter-twin estimated weight discordance >35%, and reverse end-diastolic umbilical artery flow or abnormal ductus venosus (absent/reverse a-wave).

**Table 2:** Perinatal outcomes of monochorionic twin pregnancies with type II or III Doppler patterns according to management strategy (expectant management or laser ablation of placental vessels).

Outcome	Expectant		Laser	
	Type II, n=6	Type III, n=22	Type II, n=30	Type III, n=9
**Latency to delivery, days**	80.99 (47.04-113.96)	71.50 (36.05-119)	73.99 (45.01-137.97)	66.99 (28-108.99)
**GA at delivery (weeks)**	32.43 (26.71-37)	32.85 (27.71-37.71)	32.86 (27-38)	30.14 (26.86-36)
**<28 weeks**	1/5 (20)	1/20 (5)	1/29 (3.4)	2/7 (28.6)
**<32 weeks**	2/5 (40)	7/20 (35)	11/29 (37.9)	4/7 (57.1)
**<34 weeks**	3/5 (60)	17/20 (85)	16/29 (55.2)	5/7 (71.4)
**Birth weight**				
Overall	975 (520-2770)	1555 (650-2300)	1504 (620-3300)	1317.5 (400-3400)
Smaller twin	620 (520-760) n=3	1215 (650-1880) n=18	1105 (620-2320) n=14	770 (400-1560) n=5
Larger twin	1430 (820-2770) n=5	1723 (1200-2300) n=20	1850 (600-3300) n=28	1455 (800-3400) n=7
**Fetal death**				
Overall	4/12 (33.3)	6/44 (13.6)	18/60 (30)	6/18 (33.3)
Smaller twin	3/6 (50)	4/22 (18.2)	16/30 (53.3)	4/9 (44.4)
Larger twin	1/6 (16.7)	2/22 (9.1)	2/30 (6.7)	2/9 (22.2)
At least one live born	5/6 (83.3)	20/22 (90.9)	29/30 (96.7)	7/9 (77.8)
Two live born	3/6 (50)	18/22 (81.8)	13/30 (43.3)	5/9 (55.6)
GA at death (weeks)	23.5 (21.57-25.57)	31.71 (24.28-34.28)	24.13 (18.43-30)	25.28 (18.14-26.43)
Latency from diagnosis/LAPV to death (days)	19 (17-46)	64 (26-103)	10 (1-65)	3 (1-4)
**Neonatal death**				
Overall	3/8 (37.5)	1/38 (2.6)	7/42 (16.7)	0/12 (0)
Smaller twin	2/3 (66.7)	1/18 (5.6)	5/14 (37.7)	0/5 (0)
Larger twin	1/5 (20)	0/20 (0)	2/28 (7.1)	0/7 (0)
**Perinatal death**				
Overall	7/12 (58.3)	7/44 (14.9)	25/60 (41.7)	6/18 (33.3)
Smaller twin	5/6 (83.3)	5/22 (22.7)	21/30 (70)	4/9 (44.4)
Larger twin	2/6 (33.3)	2/22 (9.1)	4/30 (13.3)	2/9 (22.2)
At least one alive	5/6 (83.3)	20/22 (90.9)	27/30 (90)	7/9 (77.8)
Two alive	0/6 (0)	17/22 (77.3)	8/30 (26.7)	5/9 (55.6)
**Intraventricular hemorrhage ≥ grade 3**				
Overall	1/6 (16.7)	1/32 (3.1)	1/41 (2.4)	1/12 (8.3)
Smaller twin	0/1 (0)	1/15 (6.7)	1/14 (7.1)	1/5 (20)
Larger twin	1/5 (20)	0/17 (0)	0/27 (0)	0/7 (0)

Data are presented as median (minimum-maximum) or number (%).
